# Synthesis and characterization of genistein magnetic molecularly imprinted polymers and their application in soy sauce products

**DOI:** 10.1038/s41598-021-02625-0

**Published:** 2021-11-30

**Authors:** Ziqi Xie, Yunjing Luo, Zhen Na, Wei Zhang, Yufei Zong

**Affiliations:** Beijing Key Laboratory of Environmental and Viral Oncology, Faculty of Environment and Life, Beijing University of Technology, No. 100, Pingleyuan, Chaoyang District, Beijing, 100124 China

**Keywords:** Analytical chemistry, Chemical synthesis

## Abstract

In this study, a novel method based on genistein magnetic molecularly imprinted polymers (Gen-MMIPs) was developed utilizing a surface molecular imprinting technique, in which genistein was used as the template molecule and Fe_3_O_4_ was used as the carrier. The synthesis of Gen-MMIPs was characterized by using scanning electron microscopy (SEM) and transmission electron microscopy (TEM), which indicated that the diameter of the Gen-MMIPs was approximately 500 nm. Via analysis with a vibrating sample magnetometer (VSM), the saturation magnetization of Gen-MMIPs was determined to be 24.79 emu g^−1^. Fourier transform infrared (FT-IR) spectroscopy showed that polymer groups were on the surface of the magnetic carrier. Adsorption experiments suggested that the genistein adsorption capability of Gen-MMIPs was 5.81 mg g^−1^, and adsorption equilibrium was achieved within 20 min. Gen-MMIPs as dispersive solid-phase extraction (dSPE) adsorbents combined with HPLC were used to selectively separate genistein in soy sauce samples, and the recoveries ranged from 85.7 to 88.5% with relative standard deviations (RSDs) less than 5%, which proved that this method can be used for the detection of genistein residues in real samples.

## Introduction

Genistein is an important bioactive component that exists in many soybean plants. Previous reports have shown that genistein has a series of pharmacological properties, such as anticancer^1^ properties, osteoporosis prevention^2^ and postmenopausal discomfort reduction^3^. However, the content of genistein in real products is extremely low. Thus, it is difficult to detect genistein in complex practical samples, which limits its application.

Several methods have been reported to determine genistein, including mass spectrometry^4^, liquid chromatography^5^, gas chromatography^6^, and an enzyme-linked immunosorbent assay^7^. Although these methods can effectively identify genistein, real samples exhibit highly complex matrices, so the analysis of genistein requires sample pretreatment methods. Sample pretreatment methods currently include solid-phase extraction (SPE)^8^, solid-phase microextraction (SPME)^9^, liquid–liquid extraction (LLE)^5^, and supercritical fluid extraction (SFE)^10,11^, but these methods can exhibit low selectivity and sensitivity. Therefore, it is necessary to develop a method with good stability, high selectivity, and high sensitivity to detect genistein in real samples.

**Figure 1:**
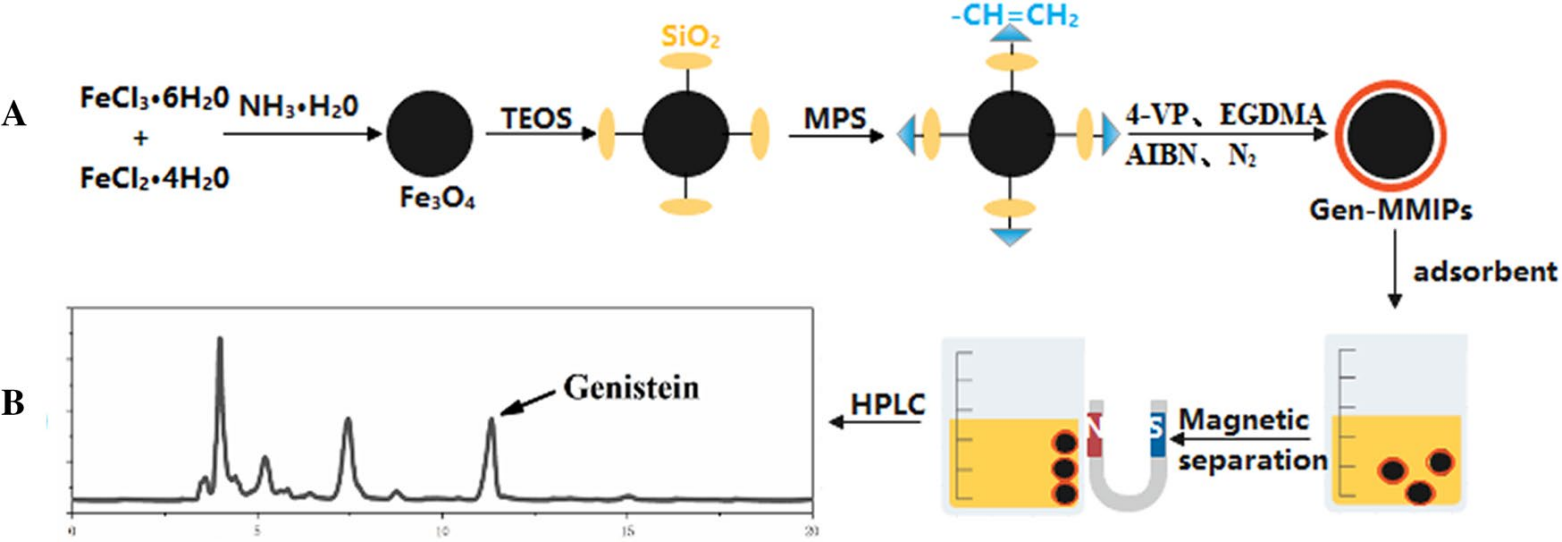
Synthesis and detection of Gen-MMIPs.

Molecularly imprinted polymers (MIPs) have been synthesized as new innovative sorbents^12–14^ and possess excellent characteristics, such as low cost, ease of preparation, excellent reusability and high selectivity^15^. Due to their excellent adsorption performance, MIPs have attracted increasing attention^16^. Compared to classical MIPs, magnetic molecularly imprinted polymers (MMIPs) have advantages such as a larger specific surface area, unique paramagnetism and better specificity and adsorption performance than ordinary polymers^17,18^. The synthesis principle of MMIPs is shown in Fig. 1A. MMIPs can be used as an adsorbent for dispersive solid-phase extraction (dSPE) and can be directly dispersed in the sample, and the target compound is extracted and enriched in the solution. After reaching adsorption equilibrium, MMIPs can be quickly separated from the solution by applying an external magnetic field^19^, which simplifies the experimental operation and reduces the use of organic reagents, thereby achieving rapid separation and enrichment of the target compound^20,21^. Therefore, this study aimed to design and synthesize a magnetic molecularly imprinted column that can specifically recognize genistein and establish a detection method for genistein in a complex matrix.

## Materials and methods

### Reagents and chemicals

Genistein, daidzein, 4-vinylpyridine (4-VP), azobisisobutyronitrile (AIBN), 3-aminopropyltriethoxysilane (APTES), and ethylene glycol dimethacrylate (EGDMA) were purchased from Sigma-Aldrich (Steinheim, Germany). 3-Trimethoxysilyl-propyl-methacrylate (MPS), tetraethoxysilane (TEOS), iron(III) chloride hexahydrate (FeCl_3_·6H_2_O), and iron(II) sulfate heptahydrate (FeSO_4_·7H_2_O) were purchased from Aladdin (Shanghai, China). Acetonitrile (HPLC grade) was purchased from Merck (Darmstadt, Germany). All other reagents and chemicals were of analytical grade or higher.

### Instruments

The morphologies of the samples were measured by scanning electron microscopy (SEM, S-4800, Hitachi, Japan) and transmission electron microscopy (TEM, Tecnai G2 F30, FEI, USA). The functional groups on the surface of the polymers were detected using a Fourier transform infrared (FT-IR) spectrometer (Nicolet 6700, ThermoFisher, USA). The magnetic properties were measured using a vibrating sample magnetometer (VSM, MPMS-3, Quantum Design, USA) at room temperature.

### Preparation process

#### Synthesis of Fe_3_O_4_ nanoparticles

Fe_3_O_4_ nanoparticles were synthesized via coprecipitation^22,23^. FeCl_2_·4H_2_O (0.88 g) and FeCl_3_·6H_2_O (2.35 g) were dissolved in 40 mL of ultrapure water. The mixture was heated to 70 °C, and 20 mL of 25% ammonia was added. Then, the reaction was maintained under constant stirring at 70 °C for 30 min. The products were collected by an external magnetic field and washed five times with ethanol. Finally, the products were dried in a vacuum at 60 °C for 12 h.

#### Synthesis of Fe_3_O_4_@SiO_2_

Fe_3_O_4_ nanoparticles (500 mg) were dispersed into 250 mL of ethanol/water (8:2, V/V) under ultrasonication for 30 min. Then, 10 mL of 25% ammonia and 10 mL of TEOS were added sequentially under nitrogen protection. The solution was then stirred at 250 rpm for 12 h at room temperature. The resultant nanoparticles were washed three times with ethyl alcohol and water after isolation by an external magnetic field. Finally, the obtained products were dried under vacuum at 60 °C for 24 h.

#### Synthesis of Fe_3_O_4_@SiO_2_–CH_2_=CH_2_

For vinyl modification, Fe_3_O_4_@SiO_2_ (500 mg) was dispersed into 200 mL of acetic acid/water (1:9, V/V) in the presence of 5 mL MPS. Next, the mixture was stirred for 24 h at room temperature. The products were magnetically separated and rinsed several times with ethanol and ultrapure water. Finally, the products were dried under vacuum at 60 °C for 12 h.

#### Synthesis of Gen-MMIPs and Gen-MNIPs

Genistein (1 mmol) and 4-VP (4 mmol) were dissolved in a three-necked flask with 40 mL of acetonitrile and stored in the refrigerator for 12 h. Then, 20 mmol of EGDMA, 0.12 mmol of AIBN, and 200 mg of Fe_3_O_4_@SiO_2_–CH_2_=CH_2_ were added to the above solution. Next, the mixture was heated to 60 °C and stirred at 300 rpm under a nitrogen atmosphere for 24 h. Gen-MMIPs were collected through magnet separation and washed with methanol/acetic acid solution (9:1, v/v) to remove the template until genistein could not be detected by UV–Vis spectrophotometry at 260 nm. Finally, the Gen-MMIPs were dried in a vacuum oven at 60 °C for 24 h. Figure 1A illustrates the preparation process of Gen-MMIPs.

Genistein magnetic nonimprinted polymers (Gen-MNIPs) were prepared under the same conditions without adding genistein.

### Adsorption experiments

#### Kinetic adsorption experiments

Gen-MMIPs and Gen-MNIPs (20 mg) were added to genistein solution (50 mg L^−1^) and incubated at different time intervals (0–60 min) at room temperature with constant shaking. The supernatant was separated by an external magnetic field, and the concentration of genistein in the supernatants was measured by HPLC–DAD at 260 nm.

#### Isotherm adsorption experiments

Gen-MMIP or Gen-MNIP particles (20 mg) were dispersed in a 5 mL centrifuge tube containing 2.0 mL of genistein solutions of various concentrations within the range of 10–100 mg L^−1^. The centrifuge tube was shaken for 20 min at room temperature, and the samples were separated by an external magnet. The free concentration of genistein in the supernatant solutions was determined using HPLC–DAD at 260 nm. The adsorption amount of genistein on Gen-MMIP particles (Q) was calculated according to Eq. (1) ^24^ in triplicate, with data reported as the mean values:

1 $$ Q_{e} = \left( {C_{0} - C_{e} } \right)V/m $$

where Q_e_ (mg g^−1^) is the equilibrium adsorption capacity; C_0_ and C_e_ are the initial and equilibrium contents (mmol L^−1^) of genistein, respectively; V (mL) is the volume of genistein solution; and m (g) is the mass of Gen-MMIPs or Gen-MNIPs.

#### Selectivity study

Daidzein was selected as a structural analogue to evaluate the selectivity of Gen-MMIPs. Gen-MMIPs or Gen-MNIPs (20 mg) were added to a 5 mL mixed solution in which the genistein and daidzein concentrations were 50 mg L^−1^ each, and the mixtures were shaken at 25 °C for 20 min. Then, genistein and daidzein in the supernatant were detected using HPLC–DAD.

### Analysis of genistein in real samples

Soy sauce samples were purchased from a local market. Soy sauce samples (2 mL) without spiking or spiking with genistein standard solutions (2 mL; 10, 20, or 40 mg L^−1^) were extracted using acetonitrile (20 mL) for 30 min. Then, the solutions were centrifuged and filtered, and the extract solutions were obtained. Gen-MMIPs were added to the extract solution in a 50 mL centrifuge tube. The centrifuge tube was shaken at room temperature for 1 h. A magnet was used to separate Gen-MMIPs from the solution, and the supernatant was collected in a 50 mL centrifuge tube and measured by HPLC–DAD to determine the amounts of genistein. Then, genistein was eluted with 3 mL of methanol/acetic acid (9:1, v/v) for 30 min, and the adsorbent was separated by a magnet. The solution containing the template molecule genistein was dissolved in 2 mL of acetonitrile solvent, and the recovery amount of genistein was determined by HPLC–DAD at 260.0 nm. All tests were performed five times with data reported as the mean values.

## Results and discussion

### Characterization

#### FT-IR analysis

**Figure 2:**
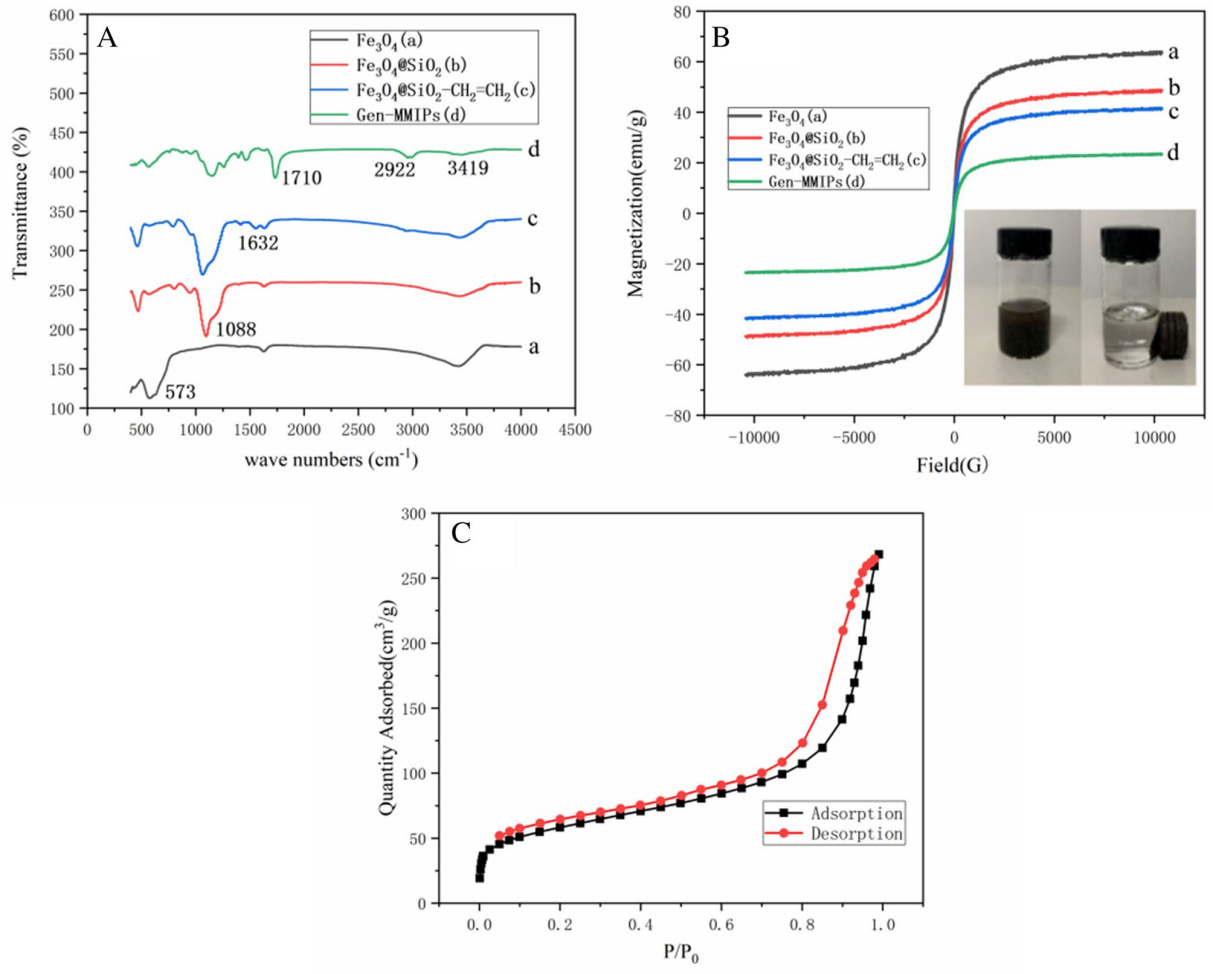
(**A**) FT-IR spectra of Fe_3_O_4_ (a)_,_ Fe_3_O_4_@SiO_2_ (b), Fe_3_O_4_@SiO_2_–CH_2_=CH_2_ (c), Gen-MMIPs (d). (**B**) VSM of Fe_3_O_4_ (a)_,_ Fe_3_O_4_@SiO_2_ (b), Fe_3_O_4_@SiO_2_–CH_2_=CH_2_ (c), Gen-MMIPs (d). (**C**) N_2_ adsorption–desorption isotherm analysis of Gen-MMIPs.

The FT-IR spectra of Fe_3_O_4_ (a)_,_ Fe_3_O_4_@SiO_2_ (b), Fe_3_O_4_@SiO_2_–CH_2_=CH_2_ (c), and Gen-MMIPs (d) are displayed in Fig. 2A. The four samples had the same adsorption peak at 3419 cm^−1^ due to the stretching vibration of O–H^24^, and the peak at 573.0 cm^−1^ characterized the Fe–O stretching vibration of Fe_3_O_4_ nanoparticles. In comparison with the curve of Fe_3_O_4_, the new characteristic peaks at 1088 cm^−1^ were attributed to the Si–O stretching vibration, indicating that SiO_2_ was coated on the surface of Fe_3_O_4_ nanoparticles. The absorption band at 1632 cm^−1^ demonstrated the existence of C=C, which proved that vinyl was synthesized on the surface of Fe_3_O_4_@SiO_2_. The absorption bands at 1710 cm^−1^ and 2922 cm^-1^ were due to the stretching vibrations of C=O and C–H in EGDMA, respectively^25^, which confirms that the MMIP layer was polymerized on the surface of Fe_3_O_4_@SiO_2_–CH_2_=CH_2_.

#### Evaluation of magnetism

The magnetic properties of the materials were characterized by a VSM. The magnetic intensity of the synthesized materials decreased with increasing polymer shell structure (Fig. 2B). The four magnetic hysteresis loops were all symmetrical to the origin, indicating that the materials were superparamagnetic^26^. The saturation magnetization of Fe_3_O_4,_ Fe_3_O_4_@SiO_2_, Fe_3_O_4_@SiO_2_–CH_2_=CH_2_, and Gen-MMIPs was 63.92, 48.77, 41.62, and 27.79 emu g^−1^, respectively. The saturation magnetization decreased each time due to the shielding effect of the coated polymer films^21,24^. Gen-MMIPs can homogeneously disperse in aqueous solutions and can be separated rapidly in solution by a magnetic field (Fig. 2B).

#### BET analysis

BET analysis was carried out to estimate Gen-MMIPs. The results of the nitrogen adsorption–desorption experiment are shown in Fig. 2C, and the general shape of the isotherm indicated the existence of micropores and macrospores^27^. The surface area of Gen-MMIPs was 207.145 m^2^ g^−1^, as calculated by Brunauer–Emmett–Teller (BET) theory. The average pore diameter was 8.03 nm. The results indicated that the specific surface area of Gen-MMIPs was sufficient to adsorb genistein.

#### TEM characterization

**Figure 3:**
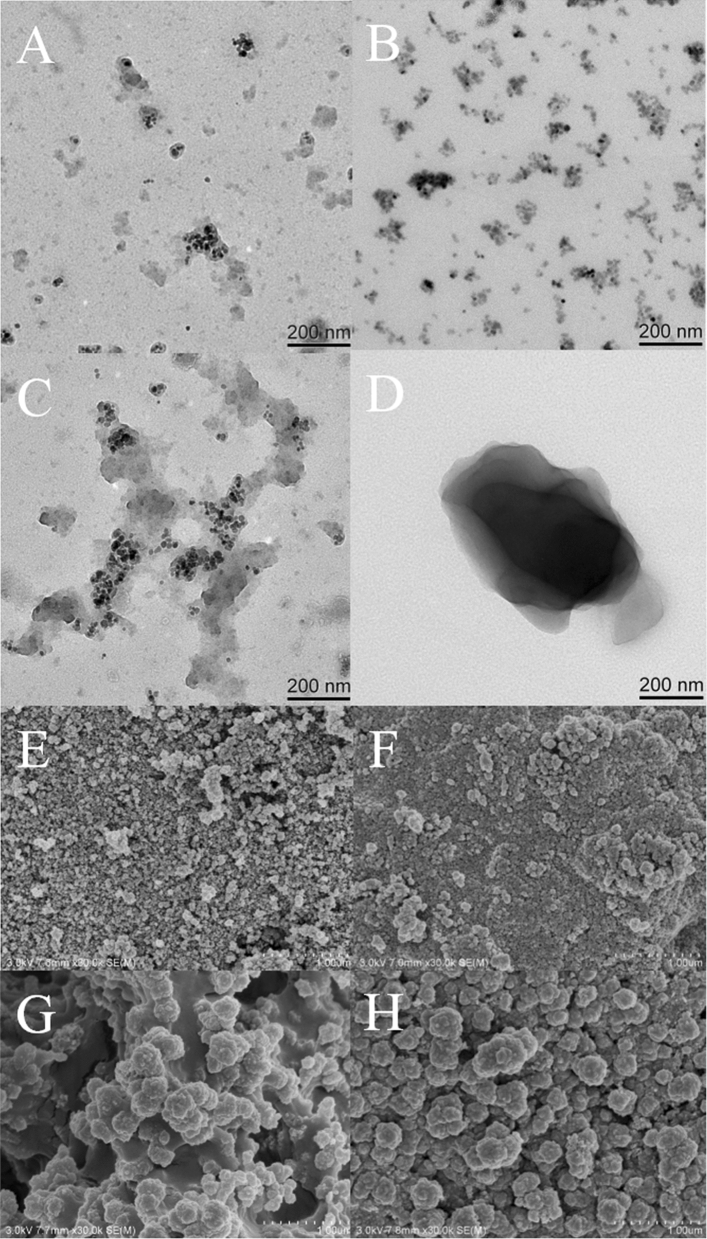
TEM images of the Fe_3_O_4_ (**A**), Fe_3_O_4_@SiO_2_ (**B**), Fe_3_O_4_@SiO_2_–CH_2_=CH_2_ (**C**) and Gen-MMIPs (**D**). SEM images of the Fe_3_O_4_ (**E**), Fe_3_O_4_@SiO_2_ (**F**), Gen-MNIPs (**G**) and Gen-MMIPs (**H**).

The morphologies of Fe_3_O_4_, Fe_3_O_4_@SiO_2_, Fe_3_O_4_@SiO_2_–CH_2_=CH_2_, and Gen-MMIPs were characterized by TEM. The diameters of Fe_3_O_4_ nanoparticles (Fig. 3A), Fe_3_O_4_@SiO_2_ (Fig. 3B), Fe_3_O_4_@SiO_2_–CH_2_=CH_2_ (Fig. 3C) and Gen-MMIPs (Fig. 3D) were approximately 10, 20, 50 and 500 nm, respectively. Compared with Fe_3_O_4_ and Fe_3_O_4_@SiO_2_, Fe_3_O_4_@SiO_2_–CH_2_=CH_2_ had a larger area and more layers of imprinted material, which indicated that vinyl was modified on the surface of Fe_3_O_4_. After the MIPs were coated, the volume of Gen-MMIPs increased significantly, and the morphologies of the Gen-MMIPs had a uniform distribution of core–shell structures. The core–shell structure of Gen-MMIPs was beneficial for the mass transfer between the solution and the surface of Gen-MMIPs.

#### SEM characterization

Figure 3E shows that the size of Fe_3_O_4_ was approximately 10 nm, which was consistent with the TEM characterization results. Compared with the Fe_3_O_4_ particles, hydrolytic TEOS coated the surface of Fe_3_O_4_ to form smoother particles (Fig. 3F). Both Gen-MMIPs and Gen-MNIPs exhibited relatively spherical structures and good dispersibility, which would provide high specific surface areas and superior structures to facilitate the mass transfer of genistein. The Gen-MNIP micrograph showed a less porous surface than that of the Gen-MMIPs, which was attributed to omitting the template during the polymerization process (Fig. 3G). Compared to Gen-MNIPs, Gen-MMIPs revealed a porous morphology and possessed more binding sites, which was beneficial for the adsorption and release of template molecules (Fig. 3H).

### Adsorption experiments

#### Adsorption kinetics

**Figure 4:**
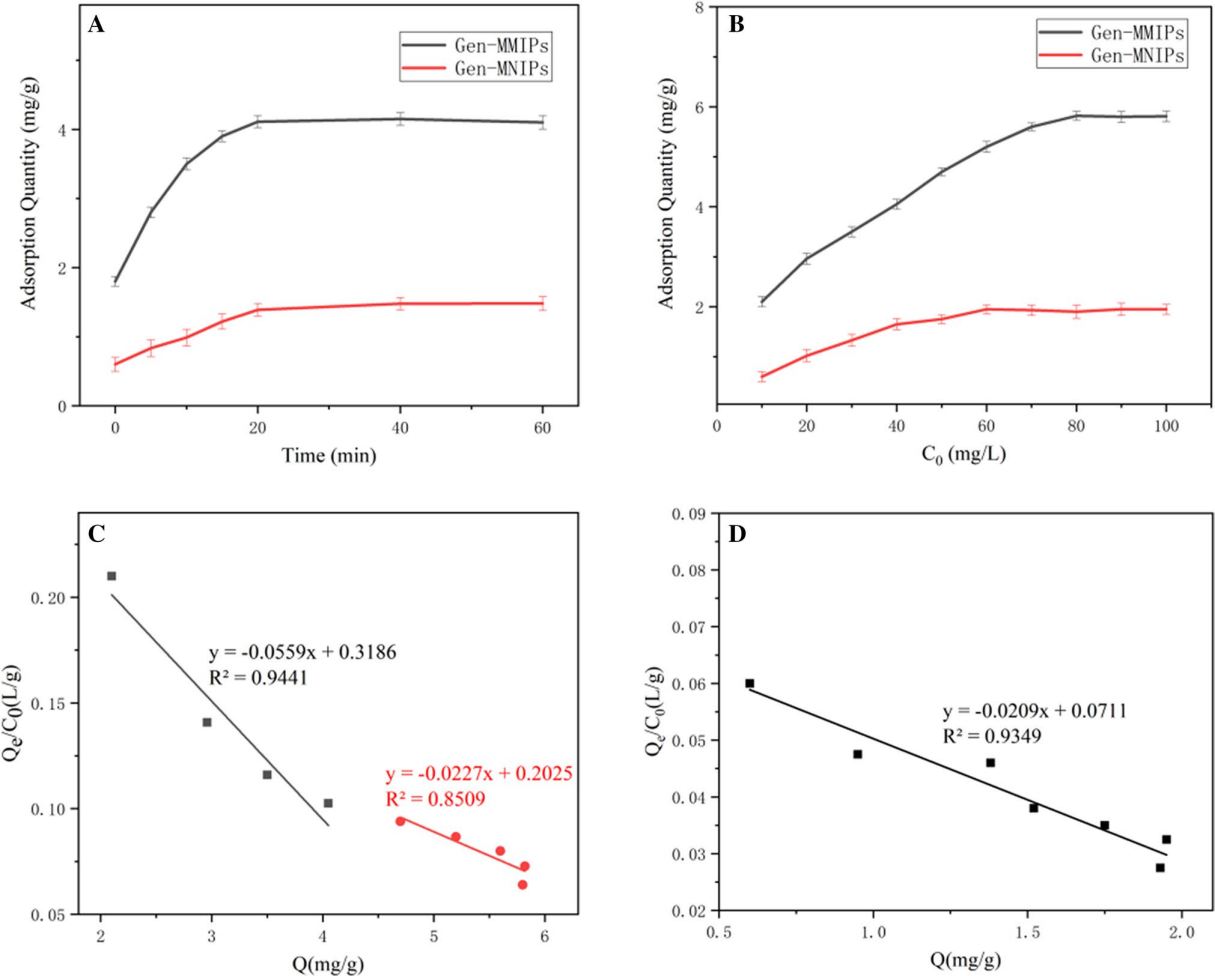
(**A**) Adsorption kinetic of Genistein on Gen-MMIPs and Gen-MNIPs, (**B**) adsorption isotherms of Genistein on Gen-MMIPs and Gen-MNIPs, (**C**) Scatchard plots of Gen-MMIPs and (**D**) Scatchard plots of Gen-MNIPs.

The adsorption kinetic curves of genistein absorbed on Gen-MMIPs and Gen-MNIPs are displayed in Fig. 4A, which shows that the adsorption capacity increased with increasing adsorption time and reached equilibrium at approximately 20 min; therefore, 20 min was selected as the optimum adsorption time because the recognition sites on the surface of Gen-MMIPs were occupied completely. The maximum adsorption amounts of Gen-MMIPs and Gen-MNIPs were 4.11 and 1.48 mg g^−1^, respectively.

#### Adsorption isotherms

The isothermal adsorption curves of Gen-MMIPs and Gen-MNIPs for genistein were estimated for different genistein concentrations (10–100 mg L^−1^). As illustrated in Fig. 4B, when the concentration of genistein was below 80 mg L^−1^, the adsorption capacity increased obviously with increasing genistein concentration. The adsorption capacity became saturated when the concentration of genistein exceeded 80 mg L^−1^. The equilibrium adsorption capacity of genistein on Gen-MMIPs was 5.81 mg g^−1^, 2.98 times higher than that of Gen-MNIPs (1.95 mg g^−1^). The genistein adsorption capacity of Gen-MMIPs was much higher than that of Gen-MNIPs at the same initial concentration. The results indicated that Gen-MMIPs possess more imprinted sites on the surface than Gen-MNIPs, which proved that Gen-MMIPs were more specific to genistein. Moreover, the equilibrium adsorption capacity of genistein by Gen-MMIPs was 5.81 mg g^−1^, which was higher than the 3.5 mg g^−1^ adsorption capacity of macroporous resins^28^ (Table S1).

The Scatchard equation was adopted to assess the binding ability of the Gen-MMIPs and Gen-MNIPs and is shown below as Eqs. (2) and (3) ^27^.

2 $$ Q_{e} /C_{e} = \left( {Q_{m} - Q_{e} } \right)/K_{d} $$

3 $$ K_{\alpha } = 1/K_{d} $$

where Q_e_ is the equilibrium adsorption capacity (mg g^−1^), C_e_ is the genistein concentration at equilibrium (mg mL^−1^), Q_m_ is the apparent maximum binding amount (mg g^−1^), K_d_ is the equilibrium dissociation constant (g L^−1^), and K_a_ is the equilibrium binding constant (L g^−1^) that reflects the affinity of binding sites. As shown in Fig. 4C, the Scatchard plot of Gen-MMIPs was two straight lines, which indicated that Gen-MMIPs have two kinds of specific binding sites to genistein—high affinity (Ka = 0.0559) and low affinity (Ka = 0.0227). The Gen-MNIP plot showed one straight line (Fig. 4D), which illustrated that there was one kind of binding site in Gen-MNIPs (Ka = 0.0209). In contrast to Gen-MNIPs, Gen-MMIPs had additional specific affinity sites, so the affinity of binding sites in Gen-MMIPs was higher than that in Gen-MNIPs.

#### Selectivity of the gen-MMIPs

**Figure 5:**
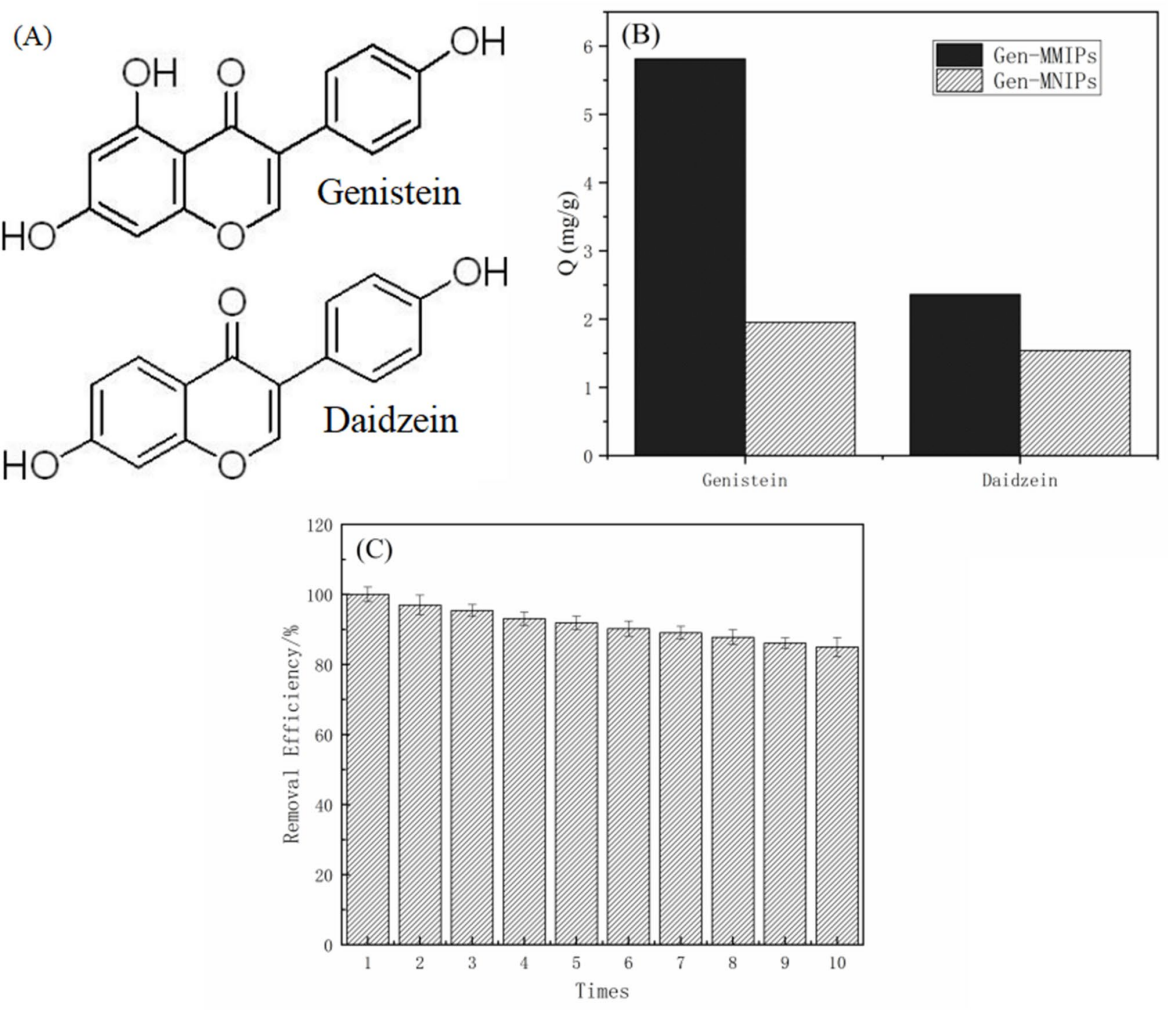
(**A**) The structures of genistein and daidzein, (**B**) selective adsorption of Gen-MMIPs and Gen-MNIPs for genistein and daidzein. (**C**) Adsorption of Genistein by Gen-MMIPs in ten adsorption–desorption cycles.

To investigate the selectivity of Gen-MMIPs towards genistein, daidzein was selected as a potential structural analogue, as shown in Fig. 5A. The adsorption capacities of Gen-MMIPs and Gen-MNIPs for genistein and daidzein are displayed in Fig. 5B. It was obvious that the genistein adsorption capacity of Gen-MMIPs was much higher than that of Gen-MNIPs. The genistein adsorption capacity of Gen-MMIPs (5.81 mg g^−1^) was approximately 2.4 times that of daidzein (2.45 mg g^−1^), which proved that Gen-MMIPs could possess more surface imprinted sites for recognition of genistein.

As shown in Fig. 5B, there was no obvious adsorption difference of Gen-MNIPs for genistein and daidzein, which suggested that Gen-MNIPs did not have the specific recognition sites that existed on their surface. Additionally, the selectivity properties of Gen-MMIPs and Gen-MNIPs towards genistein were further evaluated by imprinting factor (α) and selectivity factor (β) determination^27,29^, which were calculated according to Eqs. (4) and (5), respectively.

4 $$ \alpha = Q_{MIP} /Q_{NIP} $$

5 $$ \beta = \alpha_{Gen} /\alpha_{Dad} $$

where Q_MIP_ and Q_NIP_ (mg g^−1^) are the adsorption capacities of Gen-MMIPs and Gen-MNIPs, respectively, and α_Gen_ and α_Dad_ factors are the imprinting factors for the template (genistein) and analogue (daidzein). The imprinting factors (α) were calculated as 2.98 and 1.39 for genistein and daidzein, respectively. The selectivity factor (β) was calculated as 2.13. The results demonstrated that the obtained Gen-MMIPs exhibited good selectivity for genistein.

#### Reusability

The reusability of MIPs is an important property for application. The reusability of the Gen-MMIPs is depicted in Fig. 5C. Compared with the initial adsorption capacity, after ten adsorption-elution cycles, the adsorption capacity of Gen-MMIPs was 85% of the initial adsorption capacity. This low loss in capacity was attributed to the imprinted sites of the Gen-MMIPs being very stable and not destroyed during the process of adsorption-elution cycles.

### Analysis of spiked food samples

To evaluate the applicability of the synthesized Gen-MMIPs in real sample analysis using HPLC–DAD, soy sauce samples were pretreated according to the method in “Analysis of genistein in real samples”. The procedures used for Gen-MMIPs are shown in Fig. 1B.

#### HPLC conditions

Chromatographic analysis was performed on an HPLC–DAD system (Waters 2695, Waters Technologies, USA) with a C18 column (SunFire RM C18, 4.6 mm × 150 mm, 5 µm). The mobile phase was acetonitrile/0.1% phosphoric acid aqueous solution (5:5, V/V) at a flow rate of 1.0 mL min^−1^ at 25 °C. Spectra were monitored at 260 nm. Sample solutions were filtered through a nylon 0.22 μm filter before detection.

#### Analytical validation

Based on signal-to-noise (S/N) ratio, a limit of detection (LOD) yielding an S/N ratio of 3 and a limit of quantification (LOQ) yielding an S/N ratio of 10 were calculated to be 0.39 mg L^−1^ and 1.3 mg L^−1^, respectively. Recovery and relative standard deviation (RSD) were assessed at three levels. Reproducibility experiments (intraday, interday, interbatch and intrabatch) were performed, the results of which are shown in Table 1.

**Table 1:** Recovery and RSD for developed MISPE-HPLC method.

Spiked (mg L^−1^)	Detection (mg L^−1^)	Recovery (%) (n = 3)	RSD (%) (n = 3)	Reproducibility (RSD) (%)			
				*Intra-day (n = 5)	*Inter-day (n = 5)	Inter-batch (n = 4)	Intra-batch (n = 4)
10	8.57	85.7	2.1	2.3	3.3	4.9	4.1
20	17.42	87.1	3.0	2.6	2.9	4.6	3.8
40	35.4	88.5	2.5	3.0	3.5	5.3	4.7

*Intra-day (0, 2, 4, 8, 24 h), Inter-day (5 consecutive days).

#### Chromatograms of actual samples

To further validate the performance of Gen-MMIPs, real samples spiked with genistein were assessed by applying the optimum MMIP–HPLC–DAD method.

**Figure 6:**
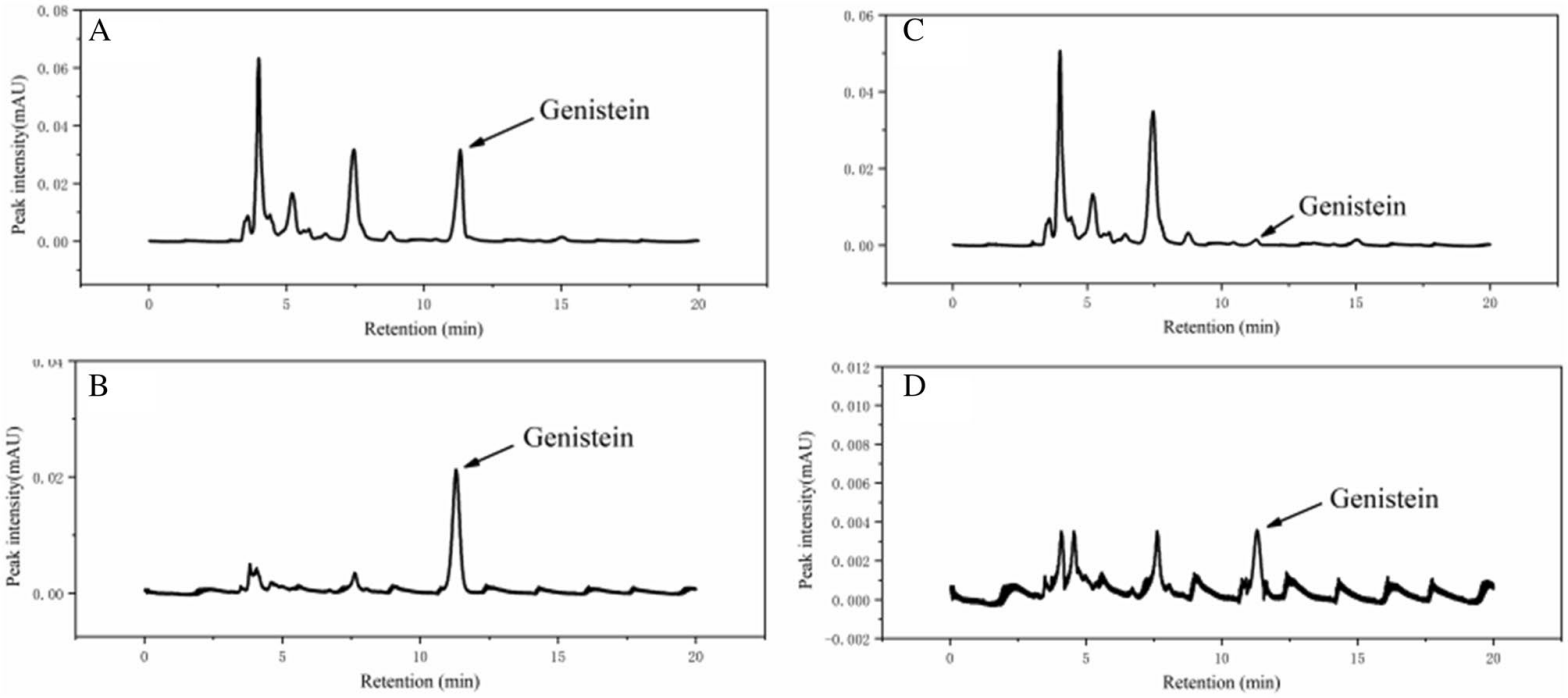
Chromatogram of soy sauce samples by Gen-MMIPs. Spike real samples (40 mg L^−1^) without enrichment (**A**); spike real samples with enrichment (**B**); real samples without enrichment (**C**) and real samples with enrichment (**D**).

The spiked sample chromatogram is shown in Fig. 6A. In addition to the absorption peak of genistein at 11 min, more than 10 peaks were observed, which showed that the actual sample had a complex matrix, and genistein accounted for a small proportion. After enrichment by Gen-MMIPs (Fig. 6B), the peak of genistein was observed without significant change; at the same time, the impurity peaks decreased obviously.

As shown in Fig. 6C, the peak of a soy sauce sample (non-spiked) at 11 min was very small, and it was difficult to identify genistein. After enrichment by the Gen-MMIPs (Fig. 6D), the content of genistein increased from 1.49 to 29.24%. Compared with existing materials such as macroporous resin, Gen-MMIPs synthesized in this study had better purification efficiency. Liu et al.^30^ separated genistein by ADS-5 resin and the genistein content reached 15.16%, while Gen-MMIPs increased the genistein content to 29.34% (Table S1). Therefore, the results show that most impurities were successfully removed by Gen-MMIPs, but there were still a few impurities in the solution after enrichment, which was caused by the physical adsorption of Gen-MMIPs. The genistein recovery of the spiked sample was 85.7–88.5%, which demonstrated that Gen-MMIPs had excellent specificity for genistein and could extract genistein in actual samples.

## Conclusions

In this study, the new adsorbent material Gen-MMIPs for genistein isolation and analysis was successfully synthesized by surface molecular imprinting. The obtained Gen-MMIPs were characterized by SEM, TEM, FT-IR and VSM. The adsorption isotherms were investigated, and the binding characteristics were found to match the Scatchard models. The Gen-MMIPs showed higher molecular selectivity and adsorption capacity than Gen-MNIPs, and the kinetic genistein adsorption of Gen-MMIPs reached equilibrium in 20 min. The superparamagnetic characteristic ensured that Gen-MMIPs could be rapidly separated by applying an external magnetic field without tedious centrifugation or filtration. In addition, Gen-MMIPs had excellent reusability. The obtained Gen-MMIPs could selectively recognize and effectively extract genistein from soy sauce samples with satisfactory recoveries and reproducibility. This work provides a novel technique for the pretreatment of samples for genistein analysis and has important practical value for the separation, analysis and application of genistein.

## Supplementary Information

Supplementary Information.
