# Supplementary material for: Synthesis and characterization of genistein magnetic molecularly imprinted polymers and their application in soy sauce products
Source: Sci Rep. 2021 Nov 30;11:23183. doi: 10.1038/s41598-021-02625-0 (PMC8633317; doi:10.1038/s41598-021-02625-0)
Supplement: Supplementary file 1 — Supplementary Information. [file 41598_2021_2625_MOESM1_ESM.doc]

##### **Synthesis and characterization of genistein magnetic molecularly imprinted polymers and its application in soy sauce products**

##### Ziqi Xie, Yunjing Luo*, Zhen Na, Wei Zhang, and Yufei Zong

##### Beijing Key Laboratory of Environmental and Viral Oncology, Faculty of Environment and Life, Beijing University of Technology, No.100, Pingleyuan, Chaoyang District, Beijing, 100124, China

##### **Corresponding author:** Dr. Yunjing Luo; No.100, Pingleyuan, Chaoyang District, Beijing, 100124, China; E-mail: luoyj@bjut.edu.cn; ORCID: 0000-0002-8915-369X

# Compliance with Ethical Standards

Conflict of interest: The authors declare that they have no conflict of interest.

| Binding efficiency  Materials | Adsorption capacity  (mg/g) | Content after enrichment (%) |
| --- | --- | --- |
| Gen-MMIPs | 5.81 | 29.24 |
| Macroporous resin | 3.5 | 15.16 |

**Table S1** Comparison of bonding efficiency with other materials
